# You say ‘prefrontal cortex' and I say ‘anterior cingulate': meta-analysis of spatial overlap in amygdala-to-prefrontal connectivity and internalizing symptomology

**DOI:** 10.1038/tp.2016.218

**Published:** 2016-11-08

**Authors:** H A Marusak, M E Thomason, C Peters, C Zundel, F Elrahal, C A Rabinak

**Affiliations:** 1 Department of Pharmacy Practice, Wayne State University, Detroit, MI, USA; 2 Merrill Palmer Skillman Institute for Child and Family Development, Wayne State University, Detroit, MI, USA; 3 Department of Pediatrics, Wayne State University School of Medicine, Detroit, MI, USA; 4 Unit on Perinatal Neural Connectivity, Perinatology Research Branch, NICHD/NIH/DHHS, Bethesda, MD, USA; 5 Behavioral Neuroscience, Boston University, Boston, MA, USA

## Abstract

Connections between the amygdala and medial prefrontal cortex (mPFC) are considered critical for the expression and regulation of emotional behavior. Abnormalities in frontoamygdala circuitry are reported across several internalizing conditions and associated risk factors (for example, childhood trauma), which may underlie the strong phenotypic overlap and co-occurrence of internalizing conditions. However, it is unclear if these findings converge on the same localized areas of mPFC or adjacent anterior cingulate cortex (ACC). Examining 46 resting-state functional connectivity magnetic resonance imaging studies of internalizing conditions or risk factors (for example, early adversity and family history), we conducted an activation likelihood estimation meta-analysis of frontoamygdala circuitry. We included all reported amygdala to frontal coordinate locations that fell within a liberal anatomically defined frontal mask. Peak effects across studies were centered in two focal subareas of the ACC: pregenual (pgACC) and subgenual (sgACC). Using publicly available maps and databases of healthy individuals, we found that observed subareas have unique connectivity profiles, patterns of neural co-activation across a range of neuropsychological tasks, and distribution of tasks spanning various behavioral domains within peak regions, also known as ‘functional fingerprints'. These results suggest disruptions in unique amygdala–ACC subcircuits across internalizing, genetic and environmental risk studies. Based on functional characterizations and the studies contributing to each peak, observed amygdala–ACC subcircuits may reflect separate transdiagnostic neural signatures. In particular, they may reflect common neurobiological substrates involved in developmental risk (sgACC), or the broad expression of emotional psychopathology (pgACC) across disease boundaries.

## Introduction

The past few years have witnessed a paradigm shift in the characterization of neuropsychiatric disorders, away from categorical descriptions towards a dimensional view.^1^ This shift is due, in part, to the observations of common behavioral, neurobiological and genetic substrates shared across phenotypically related diagnoses. This is particularly true among the internalizing disorders (for example, anxiety, depression and posttraumatic stress disorder (PTSD)), which are highly comorbid and have common heritable and environmental influences.^2, 3^ These observations have prompted the search for potential transdiagnostic neural markers (for example, Goodkind *et al.*^4^), which may provide better understanding of the etiopathogenesis of internalizing psychopathology.

Central to the internalizing disorders is the altered expression and/or regulation of emotional behavior.^5^ As such, a core emotion circuitry comprised of amygdala and medial prefrontal cortex (mPFC) has become a prime translational target for understanding the neural substrates of internalizing conditions. Abnormal resting-state functional connectivity (FC) between amygdala and mPFC is repeatedly reported across studies of internalizing conditions (for example, Brown *et al.*,^6^ Roy *et al.*^7^ and Etkin *et al.*^8^) and associated risk factors, for example, family history^9^ and exposure to childhood adversity,^10^ suggesting that frontoamygdala FC may be a transdiagnostic marker of internalizing psychopathology.

Although the extant literature converges on frontoamygdala circuitry as a core neural substrate altered across internalizing, genetic and environmental risk studies, it is unclear if findings across these studies localize to the same areas of mPFC or adjacent anterior cingulate cortex (ACC). ACC and mPFC are large, heterogeneous regions. Focal subareas within these do not have uniform function,^11, 12^ cellular composition^13^ or position within neuroanatomic circuits.^14, 15^ ACC/mPFC subregions also have distinct and frequently opposing roles in emotion processing. In general, ventral regions subserve emotion regulation, whereas dorsal regions contribute to the appraisal, expression and facilitation of emotion (see Etkin *et al.*^16^). As such, abnormalities in amygdala connectivity with different ACC/mPFC subregions likely have distinct phenotypic consequences.

To test localization of findings across studies, we conducted a coordinate-based meta-analysis of neuroimaging studies reporting disruptions in resting-state FC of the amygdala with frontal regions. We used a data-driven approach to evaluate spatial localization in studies that report significant differences in frontoamygdala FC in patient or at-risk groups. We focused on resting-state FC because it is reproducible and robust to variation in experimental parameters.^17, 18, 19^ To better understand resulting meta-analytic peak effects, we evaluated their connectivity profiles in healthy individuals using FC mapping and publicly available task activation databases. We also used quantitative functional decoding to identify the distribution of tasks spanning various behavioral domains within each meta-analytic peak, also known as ‘functional fingerprints'.^20^ Finally, we assessed the studies contributing to each peak to look for common features (for example, age and diagnosis) that could inform whether the peak was a potential marker of premorbid risk vs expression of symptomology.

## Materials and methods

### Study selection

**Figure 1:**
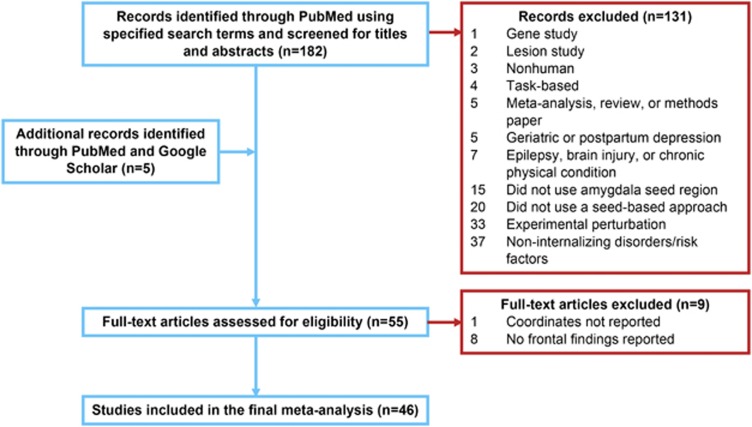
Study selection. Number of studies is given in bold letters.

The selection process occurred in multiple stages. First, we searched PubMed (www.pubmed.gov) with the keywords ‘connectivity AND (‘resting-state' OR rest OR intrinsic) AND amygdala AND (*fMRI OR ‘functional MRI' OR ‘functional magnetic' OR fc-MRI OR fcMRI) AND (PTSD OR Borderline OR internalizing OR ‘behavioral inhibition' OR stress OR adversity OR abuse OR poverty OR maltreat* OR trauma OR depress* OR anxiety OR ‘negative affect' OR ‘reward sensitivity' OR anhedonia OR mood OR bipolar or dysthymia OR ‘negative emotionality' OR ‘neuroticism') AND (‘prefrontal' OR ‘*PFC' or cingulate OR ACC OR orbitofrontal)' for the time frame up to April 2016. This search identified 182 papers. We then refined our search from the 182 identified articles assessing from the title and abstract whether the studies: (1) investigated internalizing conditions (for example, anxiety or affective disorders) or associated risk factors (for example, family history, childhood adversity, trait anxiety and behavioral inhibition); (2) examined resting-state FC of the amygdala (that is, used a seed-based approach); (3) included a group comparison between patients/at-risk individuals and matched healthy control participants, or examined risk factors on a continuum (for example, behavioral inhibition); and (4) reported coordinates in a defined stereotaxic space (Talairach or Montreal Neurological Institute). Of note, coordinates reported in Talairach space were converted into Montreal Neurological Institute for the meta-analysis.^21^ When multiple patient groups were available, we included results comparing all patients vs healthy controls. Studies were excluded if they (1) used nonhuman animals, (2) used a non-seed-based approach (for example, independent components analysis), (3) seeded regions other than the amygdala, (4) examined externalizing conditions/risk factors (for example, substance use disorder, attention deficit hyperactivity disorder and aggression) or gene polymorphisms, or (5) examined FC following an experimental perturbation (for example, psychosocial stressor, intervention and mood induction). In addition, studies examining postpartum or geriatric depression were excluded, as well as studies with comorbid epilepsy, brain injury or chronic physical condition (for example, end-stage renal disease). Of note, five additional articles were later identified through PubMed and Google Scholar by assessing similar studies that met the initial inclusion/exclusion criteria. After applying outlined exclusion/inclusion criteria (see Figure 1), 55 studies were further assessed for eligibility by reviewing the full text of the article. Nine studies were excluded after full-text review because although these studies fulfilled our initial inclusion criteria, eight studies did not report significant findings in frontal regions and one study did not report coordinate locations (Figure 1; Supplementary Table S1). Thus, 46 studies with a total of 2401 participants (*n*=893 in patient/risk group) were ultimately included in the meta-analysis (Table 1). Twelve studies evaluated heritable or temperamental risk factors (for example, negative affect and family history), 9 included individuals with major depressive disorder (MDD), 7 with anxiety disorders, 6 with PTSD, 6 environmental risk studies (for example, adversity and stress), 4 with bipolar disorder, 1 with borderline personality disorder, and 1 examined PTSD and environmental risk (early stress) within the same study. Thirty-two studies included adults and 14 included youth ages 18 and under. The majority of included studies (38) contributed more than one foci, and in total, 206 experimental foci were analyzed.

We used a data-driven approach, and included all coordinate locations reported in eligible studies that fell within an anatomically defined frontal mask (Supplementary Figure S1) comprised of the 13 frontal areas defined by the Harvard–Oxford cortical atlas (http://www.cma.mgh.harvard.edu/fsl_atlas.html): frontal operculum cortex, frontal orbital cortex, cingulate gyrus (anterior division), paracingulate gyrus, subcallosal cortex, frontal medial cortex, precentral gyrus, inferior frontal gyrus (pars opercularis), inferior frontal gyrus (pars triangularis), middle frontal gyrus, superior frontal gyrus, insular cortex and frontal pole. The mask was dilated by one voxel in all directions. Studies could contribute more than one unique frontal peak.

### ALE meta-analysis

We used the revised activation likelihood estimation (ALE) algorithm^22, 23^ to identify consistent patterns of amygdala resting-state FC changes with frontal regions. This algorithm aims to identify brain areas showing a convergence of reported coordinates across studies, which is higher than expected under a random spatial association. ALE treats reported peak coordinates, or ‘foci', as centers for three-dimensional Gaussian probability distributions that capture the spatial uncertainty associated with each focus. Width of the probability distribution is weighted based on sample size of the study from which foci were drawn, such that smaller distributions are used for larger samples and vice versa. Then, for each voxel, probabilities of all foci of a given study are aggregated to produce a modeled activation map.^24^ Modeled activation maps are combined to produce voxel-wise ALE scores, which reflect the convergence of results at each location of the brain.

Significance of convergence was assessed by comparison of ALE scores with a null distribution that includes the same number of peak foci distributed randomly throughout the brain's gray matter.^23^ Random-effects inference was applied. Resulting statistical maps show clusters where convergence between foci is greater than would be expected by chance. Statistical maps were thresholded using cluster-level family-wise error correction *P*<0.05 (cluster-forming threshold voxel-level *P*<0.001). When available, probabilistic cytoarchitectonic maps available through the SPM Anatomy toolbox^25^ were used to estimate spatial localization of results.

### Functional characterization of meta-analytic peaks

To understand the functional significance of identified meta-analytic peaks, and to test whether these represent separate frontoamygdala subcircuits, we performed three complementary analyses in healthy adults to extrapolate what differences in amygdala FC might mean in patient or at-risk groups.

#### Resting-state FC profiles

First, we derived the pattern of whole-brain resting-state FC for each meta-analytic peak. This analysis was conducted in a sample of 1000 healthy adults via www.Neurosynth.org.^26^ Results are displayed at a false discovery rate-adjusted threshold of *P*<0.01.

#### Patterns of task-related co-activation

Next, we evaluated the pattern of task-based co-activation for each peak, using meta-analytic connectivity modeling.^27^ Spherical (6 mm radii) regions of interest (ROIs) were created for each meta-analytic frontal peak, and the BrainMap database (www.brainmap.org)^28^ was searched for all functional magnetic resonance imaging (fMRI) and positron emission tomographic (PET) experiments that activated each ROI. We only considered experiments reporting stereotaxic coordinates from normal mapping studies in healthy individuals. Thus, pharmacological interventions and group comparisons were excluded. First, three-dimensional peak coordinates from peak areas that co-activate with each ROI were pooled from retrieved studies. Then, ALE meta-analysis was used to test for spatial convergence in these co-activation peaks, using similar methods as described above. ALE statistical maps were again thresholded using cluster-level family-wise error correction, *P*<0.05 (cluster-forming threshold voxel-level *P*<0.001).

#### Behavioral domains associated with activation

To further characterize observed meta-analytic peaks, we tested the distribution of tasks spanning various behavioral domains within peak regions, also known as ‘functional fingerprints'.^20^ For each ROI, we evaluated the ‘behavioral domain' meta-data from the retrieved experiments in the BrainMap database that elicited activation in that ROI (above). Behavioral domains include cognition, action, perception, emotion and interception, as well as their related subcategories (see http://brainmap.org/scribe for more information on the BrainMap taxonomy). For each domain/subcategory, the number of experiments that reported activation in each ROI was calculated. Domains/subcategories with <25 corresponding experiments are not shown.

## Results

### Meta-analysis of frontoamygdala resting-state FC across internalizing conditions and risk factors

**Figure 2:**
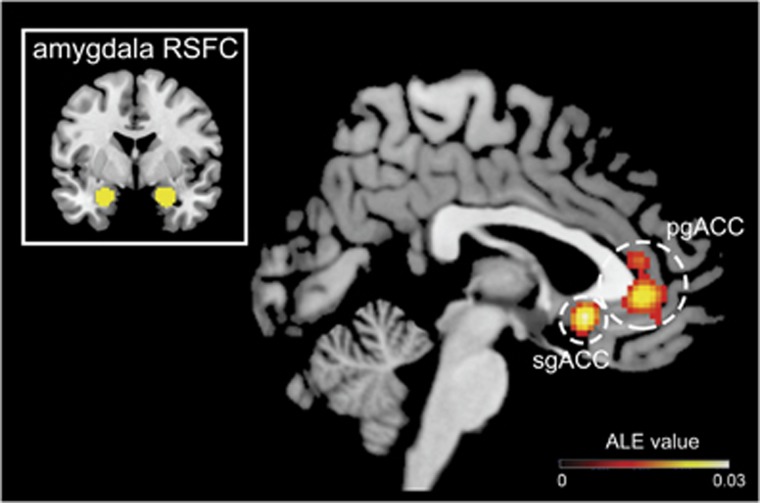
Converging evidence of disrupted amygdala functional connectivity with two separate ACC subregions across 46 internalizing, genetic and environmental risk studies. Results of coordinate-based meta-analysis that included 2401 individuals. ACC, anterior cingulate cortex; ALE, activation likelihood estimation; pgACC, pregenual ACC; RSFC, resting-state functional connectivity; sgACC, subgenual ACC.

The coordinate-based meta-analysis revealed two frontal regions, or ‘clusters', where amygdala resting-state FC was reliably altered across studies (Figure 2; Supplementary Table S2). Notably, both clusters were centered in the ACC, with limited extension into mPFC. The largest cluster was centered in bilateral pregenual ACC (pgACC), and extended into both anterior dorsal and subgenual ACC (sgACC; 8% probability in s24; see Supplementary Table S2). Hereafter, we refer to this cluster as pgACC. The second cluster was more ventral and centered in right sgACC (72% probability in s24; Supplementary Table S2).

Next, we examined the studies contributing to each cluster (Supplementary Table S2) to look for commonalities across studies. We found that studies contributing to the sgACC cluster consisted predominantly of young people ages 20 and under, with varied environmental (for example, early stress exposure) and temperamental (for example, negative affect and behavioral inhibition) risk factors. *χ*^2^ analysis suggests that studies on youth and risk factors were over-represented in the sgACC peak relative to all studies included in the meta-analysis, *χ*^2^(1)=19.92, *P*<0.001. Further, re-running the meta-analysis with the 16 studies that consisted of young people ages 20 and under yielded significant convergence in the same sgACC area (*x*=4, *y*=18, *z*=−8, ALE=0.024). The pgACC cluster, in contrast, was observed broadly across studies of anxiety disorders, affective disorders and risk factors (for example, familial risk, social inhibition and childhood adversity). Notably, although ~20% of all studies included in the meta-analysis evaluated individuals with MDD, coordinates from those studies did not show significant spatial convergence.

Across all included foci, the pattern of change was inconsistent: amygdala–frontal FC was increased in patient or at-risk groups in 50% of reported foci and decreased in 45%. Directionality was not reported for 5% of foci. We also did not find consistent patterns of change within each meta-analytic cluster. One dimension that may contribute to inconsistent findings is the application of global signal regression (GSR; Table 1). GSR is a processing step used to reduce motion-related artifact and correct the global signal in fMRI time-series data, but may result in distortions within networks and across groups.^29, 30^ When only studies that did not apply GSR were considered, we found that FC was increased in 56% of reported foci and decreased in 37%. Directionality was not specified in 7% of foci. The pattern of FC change for foci contributing to each meta-analytic peak was not more consistent when considering only studies that did not apply GSR. In addition, because FC values are typically normalized within-study, it is unclear, for example, whether increased FC reflects increased positive vs reduced negative connectivity. Specific directionality of effects (for example, increased positive FC) was reported for only 38% of included peaks.

Medication use in the study sample was also considered (Table 1). For each peak, two to three of the contributing studies reported psychotropic use in a small number of study participants (one to four). There were one to two studies that contributed to each meta-analytic peak with substantial past and/or current medication use in the patient group. Thus, observed peaks are not likely driven by medication use. We also evaluated the use of *a priori* target ROIs, which may bias results.^31^ We re-ran the meta-analysis excluding the five studies utilizing these *a priori* target ROIs (Table 1). Results were consistent with findings reported here.

Laterality of the amygdala seed region was split across study foci, with 42% reporting effects with right amygdala, 38% with left amygdala and 19% with bilateral amygdala. Laterality was also split under each meta-analytic cluster: 6 of the 12 foci contributing to the pgACC peak reported effects with left amygdala, 5 with bilateral and one with right amygdala. Three of the five foci contributing to the sgACC peak reported effects with bilateral amygdala, one with right and one with left amygdala.

### Functional characterization of ACC meta-analytic peaks

Next, we performed functional characterizations of the resulting ACC peaks in healthy individuals to infer what connectivity between amygdala and ACC may mean in patient or at-risk groups. Results may also inform whether the observed ACC peaks represent separate or overlapping brain circuits.

#### Resting-state FC profiles

**Figure 3:**
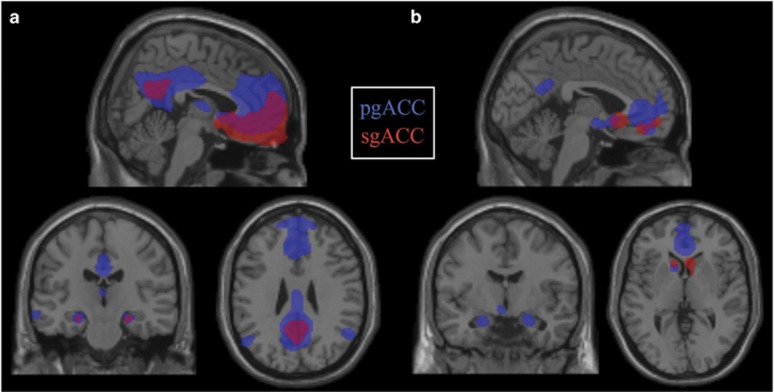
ACC meta-analytic peaks show unique patterns resting-state functional connectivity (FC) (**a**) and task-related co-activation (**b**), suggesting unique subcircuits. (**a**) Resting-state FC data in 1000 healthy individuals generated via www.Neurosynth.org, *P*<0.01 FDR corrected. (**b**) Coordinate-based meta-analysis of areas that co-activate with ACC meta-analytic peaks. A total of 971 healthy individuals contributed to pgACC and 493 to sgACC. Thresholded with cluster-level FWE correction *P*<0.05 and voxel-level, *P*<0.001. ACC, anterior cingulate cortex; FDR, false discovery rate; FWE, family-wise error; pgACC, pregenual ACC; sgACC, subgenual ACC.

We first examined patterns of resting-state FC of each ACC peak in a sample of 1000 healthy individuals. As shown in Figure 3a, whole-brain FC patterns were unique for each peak. In brief, the activity in pgACC was correlated with the activity in precuneus and posterior cingulate cortex, resembling the canonical default mode network (DMN),^32^ as well as amygdala, insula and inferior frontal gyrus, involved in the salience network.^33^ sgACC correlations were observed in local ACC regions, extending into caudate, amygdala and hippocampus, and also in precuneus and posterior cingulate cortex (Supplementary Table S3).

#### Patterns of task-related neural co-activation

We quantitatively mapped task-based co-activations for each peak using the BrainMap database. Fifty-four and 29 experiments in the BrainMap database reported activations within pgACC and sgACC ROIs, respectively. These studies consisted of 971 and 493 healthy individuals, respectively (see Supplementary Table S5 for meta-data from retrieved experiments). Using meta-analytic connectivity modeling, we found distinct patterns of co-activation clusters for each ACC peak (Figure 3b; Supplementary Table S4). In brief, pgACC was associated with co-activation clusters in caudate, posterior cingulate/precuneus, amygdala and parahippocampal gyrus. sgACC was co-activated with caudate and orbitofrontal cortex.

#### Behavioral domains associated with activation

**Figure 4:**
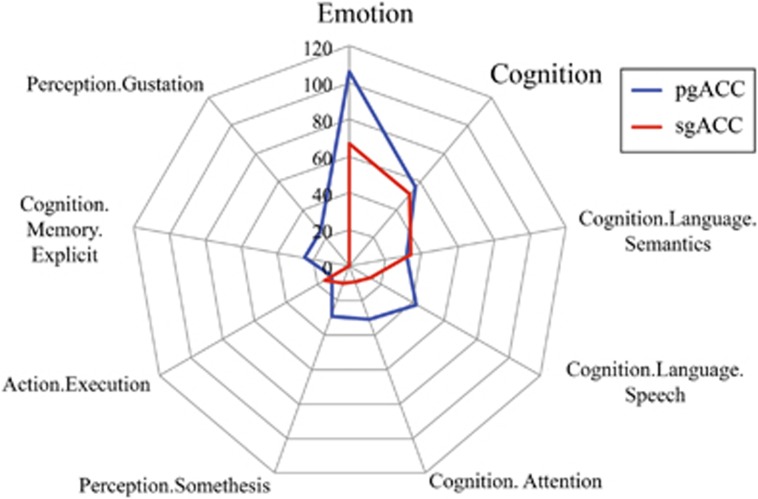
ACC meta-analytic peaks show unique functional fingerprints. Behavioral domains (number of studies) associated with activity in each ACC peak, according to studies in the www.BrainMap.org database (accessed on 2 May 2016). Behavioral domains with <25 corresponding studies are not included. A total of 971 healthy individuals contributed to pgACC and 493 to sgACC. ACC, anterior cingulate cortex; pgACC, pregenual ACC; sgACC, subgenual ACC.

To outline the functional profiles of observed peaks, we performed a functional decoding analysis based on the BrainMap meta-data. We found that activation in both pgACC and sgACC was associated with the emotion domain and, to a lesser extent, cognition (Figure 4). Relative to sgACC, pgACC activation was more likely to be associated with perception, and language and memory subcategories of cognition.

## Discussion

Altered connectivity between amygdala and frontal regions is commonly reported across a range of internalizing, genetic and environmental risk studies. Here we conducted a coordinate-based meta-analysis to test whether findings across studies localize to the same frontal subarea(s). Results converged on two focal subareas of the ACC, centered in pgACC and sgACC. Using FC analyses and publicly available databases of healthy individuals, we discovered that each peak has unique resting-state FC, functional co-activation profiles and ‘functional fingerprints'. These results suggest that observed peaks represent separate frontoamygdala subcircuits. Based on functional characterizations and the studies contributing to each peak, we assert that observed subcircuits reflect distinct transdiagnostic neural signatures. In particular, amygdala–pgACC disruptions were observed broadly in individuals across the internalizing spectrum and may thus reflect general emotional disturbance or specific symptoms that are shared across the internalizing conditions (for example, negative affect^34^). Altered amygdala–sgACC FC, in contrast, was observed almost exclusively in at-risk youth, implying a potential brain substrate of developmental vulnerability.

The largest meta-analytic cluster was centered in the pgACC, which is involved in automatic forms of emotion regulation, performing a generic negative emotion inhibitory function whenever there is a need for suppression of limbic reactivity.^35^ Explicit forms of emotion regulation occur by engaging this core circuitry (see Etkin *et al.*^16^), which is consistent with this peak's functional characterization under both cognition and emotion behavioral domains (Figure 4). Here we found that studies across the internalizing spectrum reported abnormalities in amygdala–pgACC circuitry. This raises the possibility that amygdala–pgACC circuitry is broadly involved in emotional psychopathology, or a construct that is shared across the internalizing conditions. For example, prominent models of core affect^34, 36^ emphasize that ‘loss' symptomology, or a general sense of negative affect or dysphoria (for example, feelings of sadness/withdrawal) is shared across internalizing disorders. Threat symptomology (for example, avoidance and hypervigilance), in contrast, is more specific to the anxiety disorders, and disruptions in positive affect (for example, reward deficits and anhedonia) are more specific to the mood disorders.^34^ In line with a general role of amygdala–pgACC circuitry in emotional psychopathology, reduced pgACC gray matter volume is consistently reported in meta-analyses of anxiety^37^ as well as affective disorders.^38^ Notably, FC and co-activation mapping in Figures 3 and 4 revealed strong connectivity and co-activation of the pgACC with core nodes of the DMN, including precuneus and posterior cingulate cortex. The tight coupling between pgACC and DMN may allow affective disruptions in amygdala–pgACC circuitry to integrate into self-referential processes supported by the DMN, thus propagating negative affect (see Hamilton *et al.*^39^). Connectivity was also observed between pgACC and fronto-insular regions, implicated in the salience network. Increased salience network response to negatively valenced stimuli is a consistently reported finding in MDD,^40^ suggesting a potential role for pgACC in negative emotion processing. Taken together, abnormalities in core emotion regulation amygdala–pgACC circuitry may underlie the generic negative affect dysregulation observed across internalizing conditions.

Studies contributing to the sgACC cluster consisted of environmental and temperamental risk studies (for example, childhood adversity, negative affect and behavioral inhibition) conducted predominantly in young people (ages 20 and under). Thus, disruptions in amygdala–sgACC circuitry might reflect a state of premorbid risk—a notion supported by prior research. For instance, longitudinal studies demonstrate that dysfunctional response in amygdala corresponds with genetic (that is, family history of depression) and environmental risk (that is, childhood emotional neglect^41^), and that response in sgACC predicts subsequent increases in depressive symptomology during adolescence.^42^ Broadly, sgACC is thought to subserve behavioral withdrawal and the promotion of safety behaviors.^43, 44^ Thus, early alterations in amygdala–sgACC circuitry may underlie early withdrawal behaviors that could lead to further development of internalizing symptomology. For instance, emergence of emotional psychopathology may depend on later changes in amygdala–pgACC circuitry.

Although the ALE meta-analysis identified significant spatial convergence in two areas of the cingulate cortex, a large portion (~70%) of studies did not contribute to observed meta-analytic peaks. Notably, there was particularly low spatial convergence in MDD. This is consistent with prior ALE meta-analyses in MDD and other psychiatric disorders. For example, one ALE meta-analysis in MDD^45^ reported consistent gray matter reductions (relative to healthy controls) in a similar bilateral pgACC region, with 40% of included studies contributing to this peak. In that study, and other ALE studies in psychiatric populations (for example, Chen *et al.*^46^), as low as 4% of included studies contribute to a single meta-analytic peak. Taken together, these findings suggest significant variability across studies, and particularly within MDD. Convergence within frontoamygdala circuitry might be achieved with the addition of more studies with specific patient subgroups (for example, early age of onset and recurrent). Signal dropout may also contribute to low convergence across studies, as amygdala and ventral frontal regions are highly susceptible to signal loss.^47^ Another possibility is that this variability reflects significant heterogeneity in network topology among patients. Balsters *et al.*^48^ suggest that conventional methods for generating seed regions may contribute to variable connectivity findings, as these methods do not account for heterogeneous network topology in patient groups.

Our meta-analytic results demonstrate the importance of improved anatomic specificity in reported findings. This point is not unique to the study of internalizing conditions, and there are several examples in the literature illustrating this.^16, 49, 50^ There are various means available for improving specificity in reported findings. One resource is cytoarchitectonic maps, including the widely used Brodmann areas and more recent three-dimensional multimodal brain atlases that allow registration of fMRI data into cyto-, myelo- and chemo-architectonic maps. For example, the Eickhoff–Zilles atlas distributed with SPM Anatomy toolbox^25^ and the Harvard–Oxford atlas^51^ distributed with the FSL software (http://fsl.fmrib.ox.ac.uk/fsl/fslwiki/)are increasingly used for fMRI processing and interpretation. As demonstrated here, publicly available tools and databases (for example, BrainMap and Neurosynth) can allow for better understanding of the functional profiles and circuitries in which resulting peak areas are embedded. It is encouraging that results derived from large databases (for example, coordinate-based meta-analysis and meta-analytic connectivity modeling) appear to recapitulate known cytoarchitectonic borders (see Fox *et al.*^52^).

Limitations of this work warrant mention. We focused on resting-state FC studies to circumvent variation in behavioral performance and differences in task parameters/paradigms across studies. However, there are still various experimental (for example, eyes open vs eyes closed) and analytic (for example, GSR and motion scrubbing) strategies that differ across FC studies that may have an impact on meta-analytic findings. Indeed, the field still lacks consensus on the best practices for collecting and processing resting-state FC data and, moreover, how these various approaches have an impact on observed findings. We attempted to address this by evaluating studies separately based on the use of GSR, which is known to alter resting-state FC correlations. We also provide key factors that vary between studies (Table 1), and suggest that there may be other factors contributing to variability across studies (for example, experimental or analytic methods, differences in sample characteristics, disease course and psychological state). Another consideration is that eight additional studies met criteria for inclusion, but did not report significant effects in frontal regions (Supplementary Table S1). Our goal was to examine spatial overlap in studies that do report findings in frontal regions. Future research should test the robustness of these effects using similar methodology. In addition, functional characterizations of observed meta-analytic peaks were conducted in healthy individuals, which allowed us to (1) evaluate whether peaks reflect unique brain areas that are embedded in unique circuitries, and (2) infer what behavioral consequences of altered connectivity in these areas might be. A comprehensive developmental and clinical characterization of these circuitries across ages and patient populations is warranted. Next, although we focus here on identifying focal subareas of frontal regions, there are also important subregions of the amygdala.^53, 54^ Seventeen percent of the experimental foci included in the meta-analysis reported effects of amygdala subregion(s): 17 in basolateral amygdala, 9 in centromedial and 8 in superficial. Further research is needed to understand contributions of amygdala subregion(s) to these findings, and advances in multiband and multiecho neuroimaging will make this all the more accessible.

## Conclusions

The present meta-analysis indicates that findings across internalizing, genetic and environmental risk studies converge on two focal subareas of ACC. We demonstrate that these ACC subregions have unique patterns of resting-state FC, task-related co-activation and ‘functional fingerprints', suggesting that they represent distinct frontoamygdala subcircuitries. Based on these functional characterizations and the studies contributing to each meta-analytic peak, disruptions in frontoamygdala subcircuits might reflect separate transdiagnostic neural signatures involved in developmental risk (sgACC) or the broad expression of emotional psychopathology (pgACC).

## Figures and Tables

**Table 1:** Included studies that examined internalizing conditions or risk factors and resting-state functional connectivity between amygdala and frontal regions

*#*	*First author*	*Year*	*Journal*	*Participant ages (years)*	*Total number of participants*	*% Female*	*Groups or variable of interest*	*Length of scan (min)*	*Eyes closed or eyes open*	n *on medications*	*GSR (global signal regression)?*	*Scrubbing or regressing out affected volumes?*	*Amygdala seed* definition	*Whole-brain or ROI analysis*	*Number of frontal peaks*
1	Chai	2016	*Biol Psychiatry*	8–14	43	49	Children with familial risk of MDD vs HC	6.2	Eyes open, blank screen	Not specified	No	Yes—outlier volumes regressed out	AAL atlas	Whole brain	2
2	Zhang	2016	*Prog Neuropsychopharmacol Biol Psychiatry*	38–62	66	58	PTSD vs trauma-exposed controls	8	Eyes closed	Not specified	Yes	No	AAL atlas	Whole brain	5
3	Aghajani	2016	*Hum Brain Mapp*	13–17	42	90	Sexually abused adolescents with PTSD vs controls	6	Eyes closed	3	Yes	Yes	Amunts 2005 (in SPM Anatomy toolbox)	Whole brain	4
4	Kim	2016	*Neuropsychiatr Dis Treat*	12–16	44	32	Adolescents with MDD and disruptive behaviors vs HC	12	Not specified	No current psychotropic use	No	No	AAL atlas	Whole brain	2
5	Barch	2015	*Am J Psychiatry*	7–12	105	41	Income-to-needs ratio in children	6.8	Eyes closed	Not specified	Yes	Yes	Subject-specific seeds derived from Freesurfer	Whole brain	2
6	Davey	2015	*Psychol Med*	16.5±0.5	56	45	Negative affect in adolescents	11.9	Eyes closed	1 (fluoxetine)	Yes	No	AAL atlas	ROI: BA 25 (via WFU Pickatlas)	1
7	Liu	2015	*Med Sci Monit*	13–18	46	59	Adolescents with first-episode GAD vs HC	8	Eyes closed	All med free during 2 weeks before study	Yes	no	AAL atlas	Whole brain	3
8	Nicholson	2015	*Neuropsychopharmacology*	PTSD−DS: 37±12.9 PTSD+DS: 37±12.7 HC: 32.3±11.4	89	74	PTSD±dissociative subtype (DS) vs HC	6	Eyes closed	No current psychotropic use	Not specified	Yes—outlier volumes regressed out	Amunts 2005 (in SPM Anatomy toolbox)	Whole brain	8
9	Rohr	2015	*Neuroimage*	25±2.37	43	53	Negative affectivity and task interference (ability to inhibit negative information and negative affect) in healthy adults	7.67 min & 15.33 min (1/2 of sample)	Not specified	Not specified	No	No	Harvard–Oxford atlas	Whole brain	2
10	Stoddard	2015	*Psychiatry Res*	9–18.5	53	38	Youth with BD vs vs severe mood dysregulation (SMD) vs HC	6	Not specified	42	No	Yes—outlier volumes regressed out	Amunts 2005 (in SPM Anatomy toolbox)	Whole brain	1
11	Wang	2015	*Behav Brain Res*	MDD: 32.11±11.25 HC: 33.28±8.83	60	45	MDD vs HC	8	Not specified	Not specified	Yes	No	6 mm sphere around peak atrophy voxels (−16, −6 and −16)	Whole brain	1
12	Thomason	2015	*Soc Cogn Affect Neurosci*	9–15	42	69	Trauma-exposed youth vs controls	6	Eyes closed	4 (3 trauma, 1 comparison)	No	Yes—scrubbing in secondary analysis	Amunts 2005 (in SPM Anatomy toolbox)	Whole brain and ROI in vACC	18
13	Arnold Anteraper	2014	*Brain Connect*	SAD: 24.7±6.3 HC: 25±7.5	34	53	SAD vs HC	6.4	Eyes open, fixation cross	Medication naive	No	No	Amunts 2005 (in SPM Anatomy toolbox)	Whole brain	2
14	Baeken	2014	*PLoS One*	21.7±2.5	56	100	Harm avoidance (personality dimension) in healthy adults	5	Eyes closed	None used medications	No	No	−20, −4, -15 and 22, −2, 15 (Cisler *et al.*)	Whole brain	10
15	Birn	2014	*Depress Anxiety*	22–31	27	0	Childhood adversity and PTSD symptoms in veterans	5.5	Not specified	No current med use	No	Yes—despiking	4mm spheres centered on coordinate centers provided by Talarach Daemon	Whole brain	8
16	Blackford	2014	*Biol Psychol*	18–25	40	60	Social inhibition in young adults (*n*=8 met criteria for 1 or more AD)	7	Eyes closed	No current psychotropic use	Yes	No	Amunts 2005 (in SPM Anatomy toolbox)	Whole brain	12
17	Aghajani	2014	*Cogn Affect Behav Neurosci*	40.51± 9.45	50	64	Trait neuroticism in healthy adults	7.67	Eyes closed	No current med use	Yes	No	Harvard–Oxford atlas	Whole brain	3
18	Qin	2014	*Biological Psychiatry*	7–9	76	50	Anxiety scores in children	8	Eyes closed	No current psychotropic use	Yes	Yes—scrubbing in secondary analysis	Amunts 2005 (in SPM Anatomy toolbox)	Whole brain	6
19	Brown	2014	*Neuropsychopharmacology*	PTSD: 44.1±11 Trauma-exposed controls: 40±8.9	42	24	PTSD vs trauma-exposed controls (recent military veterans)	6.3	Eyes open, fixation cross	14	No	Yes—scrubbing in secondary analysis	Amunts 2005 (in SPM Anatomy toolbox)	Whole brain	2
20	Coombs	2014	*PLoS One*	19–53	38	29	Negative affect in healthy adults	6.2	Eyes open, blank screen	No lifetime med use	Yes	No	Jeulich Histological atlas	Whole brain	1
21	Fan	2014	*Hum Brain Mapp*	21–36	18	0	Early-life stress exposure in adults	8	Eyes open, fixation cross	Not specified	No	No	AAL atlas and based on meta-analysis of emotion processing (Wager *et al.*, 2012)	Whole brain	15
22	Golkar	2014	*PLoS One*	19–46	93	57	Work-related (perceived) chronic stress in adults	8	Eyes closed	All med free during 2 months before study (except contraceptives)	No	No	5 mm spheres around (−22, −7, −18 and 22, −7, −19)	Whole brain	5
23	Hamm	2014	*Biol Mood Anxiety Disord*	AD: 13.9±3.1 HC: 14.6±3.9	55	64	Pediatric AD (GAD, social phobia and SAD) vs HC	8	Eyes open, fixation cross	All med free at the time of scan	No	No	AAL atlas based on Talairach Daemon database	Partial brain mask of mPFC, ACC, PCC and insula (AAL atlas)	5
24	Jacobs	2014	*PLoS One*	18–23	53	66	Remitted MDD vs HC	8	Eyes open	All med free during 30 days prior to study	No	Yes—scrubbing in secondary analysis	±23, −5, −19 (2.9 mm radius sphere)	Whole brain	1
25	Krause-Utz	2014	*Psychol Med*	18–45	37	100	Borderline personality disorder (with the history of interpersonal trauma) vs HC	6.25	Eyes closed	Free of medication within the past 14 days (28 on fluoxetine)	Yes (repeated analyses without)	No	±23, −4, −19 (Veer *et al.*, 2011; 4 mm radius sphere)	Whole brain	2
26	Liu	2014	*Schizophr Bull*	BD: 33±10.0 HC: 36.6±12	36	61	BD (no comorbidities) vs HC	not specified	Eyes closed	18 (bipolar patients)	Yes	No	Amunts 2005 (in SPM Anatomy toolbox)	Prefrontal mask (BA's 9–12, 24, 25, 32 and 44–47)	8
27	Pannekoek	2014	*J Child Psychol Psychiatry*	MDD: 15.4±1.5 HC: 14.7±1.5	52	88	Youth with MDD vs HC	6	Eyes closed	None used medications	Yes	No	Harvard–Oxford Subcortical Structural Probability atlas (in FSL; ±22, −6, −16)	Whole brain	3
28	Ramasubbu	2014	*Front Psychiatry*	MDD: 36.5 ±10.41 HC: 32.89±9.97	74	59	MDD vs HC	7.67	Eyes open, fixation cross	All med free at the time of scanning. Fifty-two MDD patients had been previously exposed to antidepressants	No	No	FSLView and registered to participants native fMRI image	Whole brain	9
29	Roy	2014	*Biological Psychiatry*	BI: 19.6±1 Non-BI: 19.5±0.94	38	53	Young adults with childhood history of behavioral inhibition	6	Eyes closed	No current psychotropic use	Yes	Yes—despiking	Amunts 2005 (in SPM Anatomy toolbox)	Whole brain	4
30	Singh	2014	*Bipolar Disord*	8–17	49	63	Youth with low vs high familial risk of BD	7	Eyes closed	None used medications	No	Yes—outlier volumes regressed out	Harvard–Oxford atlas	Whole brain	1
31	Zhang	2014	*PLoS One*	18–24	67	52	First-episode MDD vs HC	5	Eyes closed	Medication naive	Yes	No	AAL atlas	Whole brain	1
32	Anticevic	2013	*Biological Psychiatry*	Bipolar 1 psychosis hx: 34±10.8 Bipolar 1 no psychosis hx: 29.85±11.9 HC: 31.14±10.6	119	65	BD (1/2 with the history of psychosis; 46% with comorbid AD, 57% alcohol use, and 43% drug use) vs HC	5.25	Eyes open	57	Yes	No	Freesurfer-based segmentation	Whole brain	2
33	Carlson	2013	*Cortex*	19–23	15	60	Attentional bias to threat	5	Eyes closed	Not specified	No	No	Harvard–Oxford atlas	ROI: 6 mm sphere at ±4, 46, −4 (ACC)	8
34	Herringa	2013	*Proc Natl Acad Sci USA*	18.79±0.19	64	47	Young adults with maltreatment during childhood	7	Eyes closed	Not specified	Yes	Yes—scrubbing	4 mm rad spheres in amygdala defined by Talairach Daemon	Whole brain	3
35	Prater	2013	*Depress Anxiety*	gSAD: 25.95±5.39 HC: 25.71±7.15	37	57	gSAD vs HC	5	Eyes open, fixation cross	2 (gSAD patients; SSRIs)	Yes	No	All faces>shapes localizer confined within AAL-defined anatomical amygdala	Partial brain mask of ACC, mPFC, DLPFC and OFC (AAL atlas)	2
36	Roy	2013	*J Am Acad Child Adolesc Psychiatry*	12–17	35	66	Youth with GAD vs HC	6	Eyes open, fixation cross	No current or past use of psychotropic medication	Yes	No	Amunts 2005 (in SPM Anatomy toolbox)	Whole brain	3
37	Tahmasian	2013	*Front Human Neurosci*	MDD: 51±15 HC: 49.6±13.9	41	54	MDD vs HC	10	Eyes closed	40 (5 with antidepressant mono-therapy, 10 with dual therapy and 5 with tri-therapy)	Yes	No	Amunts 2005 (in SPM Anatomy toolbox)	Whole brain	4
38	Tang	2013	*Psychol Med*	MDD: 29.3±8.7 HC: 30.1± 8.4	58	53	MDD vs HC	6.67	Eyes closed	Treatment naive	Yes	No	AAL atlas	Whole brain	2
39	Torrisi	2013	*Bipolar Disord*	BP1: 42.1±11.4 HC: 39.8±12.6	40	50	BD (I) vs HC	7	Eyes closed	17 (bipolar patients)	No	No	Talairach Daemen and Harvard–Oxford	Whole brain	3
40	van der Werff	2013	*Psychol Med*	CEM: 39±10.3 No CEM: 37.6±9.7	88	52	Adults reporting childhood emotional maltreatment (CEM; before age 16; no physical or sexual abuse) vs non-CEM	7.6	Not specified	Not specified	Yes	No	Harvard–Oxford atlas	Whole brain	5
41	Sripada	2012	*J Psychiatry Neurosci*	21–37	29	0	PTSD vs combat-exposed controls	10	Eyes open, fixation cross	1 (trazodone as sleep aid)	Yes	No	Amunts 2005 (in SPM Anatomy toolbox)	Whole brain	3
42	Hahn	2011	*Neuroimage*	GAD: 27.7±7.2 HC: 28.6±4.3	37	46	AD (SAD, PD or both) vs HC	6	Eyes open, low-level illumination	All med free during 3 months before study	Yes	No	AAL atlas	Whole brain	3
43	Kim	2011	*Cereb Cortex*	19.± −0.9	29	72	Anxiety scores in healthy adults	7	Eyes open, ‘relax' on screen	None used medications	Yes	No	Harvard–Oxford atlas	Whole brain	2
44	Lui	2011	*Am J Psychiatry*	Nonrefractory MDD: 32±10 Refractory MDD: 33±11 HC: 35±12	108	35	MDD (nonrefractory) vs HC	6.7	Eyes closed	108	No	No	AAL atlas	Whole brain	3
45	Rabinak	2011	*Front Psychiatry*	PTSD: 30.12±7.70 Combat-exposed controls: 33.71±9.12	34	0	PTSD vs combat-exposed controls	8	Eyes open, fixation	All med free at time of scan. *N*=8 hx of psychotropic (*n*=8 SSRI and *n*=1 also taken NE-DA reuptake inhibitor, *n*=1 tricyclic, *n*=2 5-HT antagonist reuptake)	Yes	No	Walter 2003 (in MARINA software)	Whole brain	1
46	Liao	2010	*PLoS One*	SAD: 22.55±4.05 HC: 21.71±3.64	43	28	SAD vs HC	6.83	Eyes closed	All med free at the time of scan	Yes	No	AAL atlas	Whole brain	15

Abbreviations: AAL, Automated Anatomical Labeling; ACC, anterior cingulate cortex; AD, anxiety disorder; BD, bipolar disorder; DLPFC, dorsolateral prefrontal cortex; fMRI, functional magnetic resonance imaging; GAD, generalized anxiety disorder; gSAD, generalized social anxiety disorder; HC, healthy controls; med, medication; MDD, major depressive disorder; mPFC, medial prefrontal cortex; OFC, orbitofrontal cortex; PD, panic disorder; PTSD, posttraumatic stress disorder; ROI, region of interest; SSRIs, selective serotonin reuptake inhibitors; SAD, social anxiety disorder.
