# Supplementary figures and images for: You say ‘prefrontal cortex' and I say ‘anterior cingulate': meta-analysis of spatial overlap in amygdala-to-prefrontal connectivity and internalizing symptomology
Source: Transl Psychiatry. 2016 Nov 8;6(11):e944–. doi: 10.1038/tp.2016.218 (PMC5314129; doi:10.1038/tp.2016.218)

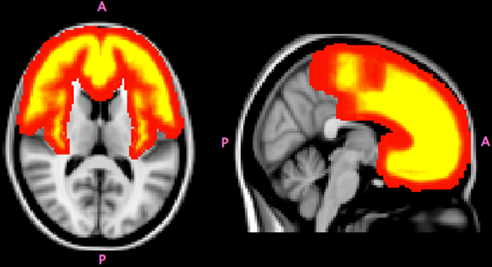

Supplement: Supplementary Figure [file tp2016218x6.tif]
